# Effect of dietary glycine and betaine on productive performance, liver health, intestinal characteristics, and stress response in aged laying hens under heat stress conditions

**DOI:** 10.5713/ab.250618

**Published:** 2025-11-10

**Authors:** Deok Yun Kim, Ryun Ha Kim, Hyun Woo Kim, Ji Hye Lee, Dong Yong Kil

**Affiliations:** 1 Department of Animal Science and Technology, Chung-Ang University, Anseong, Korea

**Keywords:** Aged Laying Hen, Betaine, Glycine, Heat Stress, Productive Performance, Stress Response

## Abstract

**Objective:**

This study aimed to investigate the effect of dietary glycine (Gly) and betaine (Bet) on productive performance, egg quality, liver health, intestinal characteristics, and stress response in aged laying hens under heat stress (HS) conditions.

**Methods:**

A total of 384 aged laying hens were allotted to 1 of 4 dietary treatments in a completely randomized design with a 2×2 factorial arrangement, including 2 supplemental levels of Gly (0% and 0.65%) and Bet (0% and 0.20%) in diets. Each treatment had 8 replicates. All hens were exposed to a cyclic HS condition at 31.7±1.7°C for 8 hour/day and 27.2±1.3°C for the remaining time during a 12-week feeding trial.

**Results:**

No main and interactive effects of dietary Gly and Bet supplementation were identified for productive performance and egg quality in aged laying hens under HS conditions. However, for the main effects, Gly supplementation decreased liver color score (p<0.01) and malondialdehyde (MDA) levels (p<0.05), while Bet supplementation also decreased liver MDA levels (p<0.05). An improvement in intestinal barrier function (p<0.01) and a decrease in feather corticosterone concentrations (p<0.01) were observed by individual and combined supplementation of Gly and Bet. However, combined supplementation of Gly and Bet showed no synergistic benefits over individual supplementation.

**Conclusion:**

Dietary supplementation of 0.65% Gly and 0.20% Bet improved liver health, intestinal barrier function, and reduced stress responses in aged laying hens under HS conditions with little interactive effects of their combined supplementation.

## INTRODUCTION

Heat stress (HS) has emerged as a considerable challenge in the poultry industry due to the recent global climate change. The negative impact of HS on productive performance, product quality, and health in poultry has been documented [1,2]. Among various poultry species, it appears that aged laying hens are highly susceptible to HS due to their decreased capacity for temperature regulation, antioxidant defense, nutrient utilization, and immune defense with age [1,3]. Moreover, these age-related physiological problems are frequently linked to impaired liver health, including enhanced hepatic fat accumulation and oxidative damage, in laying hens under HS conditions [4,5]. Therefore, the development of effective strategies to mitigate the negative impact of HS on aged laying hens is essential.

Betaine (Bet) plays a crucial role as an osmolyte that maintains cellular water balance in animals under HS conditions [6,7]. The Bet also functions as an active methyl donor in one-carbon metabolism, which is essential for various body metabolisms [7]. Moreover, dietary Bet supplementation has been reported to alleviate oxidative stress by enhancing the activities of key antioxidant enzymes, including superoxide dismutase and glutathione peroxidase, thereby reducing HS-induced oxidative damages [8]. Consequently, dietary Bet is widely used as a feed supplement to mitigate HS-induced negative outcomes in poultry [6,7].

Glycine (Gly) is typically considered a non-essential amino acid (AA) in poultry [9]. However, additional supplementation of Gly in poultry diets has gained increasing attention because Gly is a precursor molecule for the biosynthesis of creatine, heme, glutathione, bile acids, nucleic acids, and uric acid [9], and the requirement of those physiologically important molecules is likely increased under stressful conditions [2]. Furthermore, Gly can act as a biological osmolyte and methyl donor [10], serving a function similar to Bet, suggesting that Gly may be a functional AA that ameliorates the negative impact of HS in poultry [11,12]. In addition, a previous study demonstrated that dietary Gly supplementation reduced the fatty liver incidence in laying hens under HS conditions by decreasing abnormal fat retention in the liver [11].

The metabolism of Gly and Bet is closely associated because both Gly and Bet are linked with transmethylation in the body and Bet is a precursor molecule for endogenous Gly synthesis [7]. Therefore, it can be hypothesized that the physiological benefit of Gly and Bet is complementary to ameliorate the negative impact of HS in poultry. Moreover, given that aged laying hens are highly vulnerable to HS, it is also anticipated that synergistic interaction of dietary Gly and Bet combination may be significant in aged laying hens under HS conditions. However, no studies have been performed to test this hypothesis.

Therefore, the current study aimed to investigate the interactive effect of dietary supplementation of Gly and Bet on productive performance, egg quality, liver health, intestinal characteristics, and stress response in aged laying hens raised under HS conditions.

## MATERIALS AND METHODS

### Animals, diets, and experimental design

A total of 384 Lohmann Brown-Classic aged laying hens at 65 weeks of age were allotted to 1 of 4 dietary treatments in a completely randomized design with a 2×2 factorial arrangement, including 2 supplemental levels of Gly (0% and 0.65%) and Bet (0% and 0.20%) in diets. Each treatment had 8 replicates consisting of 12 cages with 1 hen per cage (24 cm×36 cm×39 cm = width×length×height). The average body weight (BW) and egg production rate of hens at the start of the experiment were 1.81 kg and 89.1%, respectively. The basal diet was prepared to meet or exceed the recommended concentrations of energy and all nutrients for Lohmann Brown laying hens (Table 1). Three treatment diets were prepared by supplementing the basal diet with 0.65% Gly (99.0%; Samin Chemical), 0.20% Bet (98.0%; Gene-biotech), or a combination of two supplements (0.65% Gly+0.20% Bet).

The concentrations of AA in the basal diet were calculated using the concentrations of standardized ileal digestible (SID) AA of individual ingredients presented in the Brazilian Tables for Poultry and Swine [13]. The supplemental level of Bet (0.20%) was determined based on previous studies [12,14], whereas the supplemental level of Gly (0.65%) was designed to increase the concentrations of SID Gly+Serine (Ser) by 50% compared with the basal diet (1.29% SID Gly+Ser). Additional alanine was supplemented to treatment diets with no Gly supplementation to equalize the concentrations of crude protein (CP) among treatment diets [12]. The analyzed concentrations of total CP, Gly, and Ser in treatment diets are presented in Table 1. Hens were provided with water and feed *ad libitum* for a 12-week feeding trial. All hens were exposed to a cyclic HS condition at 31.7±1.7°C for 8 hours per day and 27.2±1.3°C for the remaining time. The average relative humidity was 70±12.6% during the whole experiment. The HS index for laying hens was calculated based on ambient temperature and relative humidity [15]. Under the current environmental conditions, the HS index values ranged from approximately 77 to 84, indicating that aged laying hens raised in this study were exposed to severe HS conditions [15]. A 14-hour lighting schedule was maintained throughout the experiment.

### Sample collection and analysis

The experimental diets were analyzed for CP (method 984.13; [16]) using the Kjeldahl method (Kjeltec Auto System 2300 Analyzer; FOSS). The diets were also analyzed for total Gly and Ser using an L8900 amino acid analyzer (Hitachi) after hydrolysis in 6 N HCl at 110°C for 24 hours (method 999.13; [16]).

At the conclusion of the study (i.e., 76 weeks of age), 2 hens with BW close to the average BW in each replicate were selected and euthanized by CO_2_ asphyxiation. One hen was used to collect blood and tissue samples of the liver, jejunum, and feathers, whereas the other hen was used to collect the jejunal mucosa for the analysis of intestinal permeability using a Ussing chamber.

### Productive performance and egg quality

Productive performance, including hen-day egg production, egg weight, egg mass, and broken and shell-less egg production rate, was recorded daily. Feed intake (FI) and feed conversion ratio (FCR) were calculated weekly. The data for productive performance were summarized for 12 weeks of the feeding trial.

Egg quality, including eggshell color (i.e., eggshell color fan and colorimeter), egg yolk color, eggshell strength, eggshell thickness, and Haugh unit, was assessed using 12 eggs randomly collected per replicate during the last 2 days of the experiment (i.e., 6 eggs per day). The detailed procedures to measure egg quality were described in a previous study [17].

### Liver measurements

The liver attached to the body was photographed to assign a subjective fatty liver score using a scale from 1 to 5 (1 = dark red; 5 = yellowish red) for the analysis of fatty liver incidence [18]. Moreover, the objective CIE color values for lightness (L*), redness (a*), and yellowness (b*) were determined using a colorimeter (model CR-10; Konica Minolta Optics). The subjective liver hemorrhage was also scored from 0 to 5, reflecting normal liver at 0 and extensive hemorrhages at 5 [17]. A portion of the liver sample was used to analyze the concentrations of acid-hydrolyzed ether extract (AEE; method 954.02; [16]). Malondialdehyde (MDA) concentrations in the liver were also measured using a thiobarbituric acid reactive substances (TBARS) Assay Kit (Catalog No. STA-330; Cell Biolabs), following the manufacturer’s protocol.

Blood samples were also collected via heart puncture into a 6-mL serum tubes (BD Vacutainer Serum, BD) and immediately centrifuged at 3,000×g at 4°C for 15 min to obtain the serum. The serum concentrations of aspartate aminotransferase (AST) and alanine aminotransferase (ALT) as liver health indicators were measured using a Hitachi Automatic Analyzer 7020 (Hitachi).

### Intestinal measurements

Jejunal segments measuring 1–2 cm in length were collected for the analysis of intestinal morphology. Jejunal segments were flushed and fixed with 10% neutral-buffered formalin solution. The villus height (VH), crypt depth (CD), and VH:CD were measured following the method of Nari et al [19]. Data were obtained from the average values of 20 intestinal measurements. Trans-epithelial resistance (TER) values as a measure of intestinal permeability in the jejunal mucosa were determined using a dual channel self-contained Ussing chamber system (U2500; Warner Instruments). The detailed procedure was reported previously [20].

### Stress indicator

Blood samples were collected via heart puncture into 10-mL EDTA tubes (BD Vacutainer K2EBD, BD). The heterophil-to-lymphocyte ratio (H:L) as a stress indicator in the blood was analyzed using the method described by Lentfer et al [21]. The detailed procedure was reported in a previous study [11]. Flight feathers were also collected to analyze the concentrations of feather corticosterone (CORT) according to the method of Bortolotti et al [22]. The detailed procedures were reported in a previous study [23]. This analysis was performed at the BT research facility center, Chung-Ang University.

### Statistical analysis

All data were analyzed by two-way ANOVA in a completely randomized design using the PROC MIXED procedure (SAS Institute). The replicate was considered an experimental unit. The statistical model included the effect of Gly and Bet supplementation, and their interaction. The LSMEANS procedure was used to calculate treatment means. In addition, if the interaction was significant, treatment means were separated to clarify the interaction among treatment means based on the PDIFF option of SAS. The statistical significance was set at p<0.05.

## RESULTS

### Productive performance and egg quality

No main and interactive effects between dietary supplementation of 0.65% Gly and 0.20% Bet were observed for all productive performance, including hen-day egg production, egg weight, egg mass, broken and shell-less eggs, FI, and FCR in aged laying hens under HS conditions for the 12-week feeding trial (Table 2).

In egg quality, an interaction (p<0.05) was identified for eggshell color fan score owing to the observation that dietary supplementation of Bet alone did not affect eggshell color fan score, whereas dietary supplementation of Bet in Gly-supplemented diets improved (p<0.05) eggshell color fan score compared with dietary supplementation of Gly alone. However, no interactive effects of dietary supplementation of Gly and Bet were observed for other egg quality measurements, including eggshell CIE color values (L*, a*, and b*), egg yolk color, Haugh unit, eggshell thickness, and eggshell strength. Likewise, dietary supplementation of Gly or Bet as the main effects did not influence all egg quality measurements (Table 3).

### Liver health

Interactive effects of dietary supplementation of Gly and Bet were not found for all liver health measurements, including liver color score, hemorrhagic score, AEE concentrations, and MDA levels in aged laying hens under HS conditions (Table 4). However, for the main effects, dietary Gly supplementation, irrespective of Bet supplementation, decreased the liver color score (p<0.01) and MDA levels (p<0.05). Likewise, dietary Bet supplementation decreased (p<0.05) MDA levels in the liver, regardless of Gly supplementation. Nevertheless, no main effects of dietary supplementation of Gly or Bet were observed for liver hemorrhagic score and AEE concentrations as well as serum AST and ALT levels.

### Intestinal morphology and permeability

An interaction (p<0.05) between dietary supplementation of Gly and Bet was identified for CD in the jejunum of aged laying hens under HS conditions because dietary supplementation of Bet alone increased CD (p<0.05), whereas dietary supplementation of Bet in Gly-supplemented diets did not affect CD (Table 5). However, regarding the main effects, dietary Bet supplementation increased CD (p<0.05), but such an effect was not observed by dietary Gly supplementation. No interactive and main effects of dietary supplementation of Gly and Bet were found for VH and VH:CD.

Jejunal TER values as a measure of intestinal permeability were increased (p<0.05) by dietary supplementation of Bet alone but remained unaffected when dietary Bet was supplemented to Gly-supplemented diets, leading to an interaction (p<0.01). Likewise, the main effects demonstrated that dietary supplementation of Gly or Bet increased (p<0.05) jejunal TER values in aged laying hens under HS conditions.

### Stress indicators

No interaction between dietary supplementation of Gly and Bet was found for blood H:L in aged laying hens under HS conditions (Table 6). However, an interaction (p<0.01) was identified for feather CORT concentrations due to the observation that dietary supplementation of Bet alone decreased (p<0.05) feather CORT concentrations, but this effect was not observed when dietary Bet was supplemented to Gly-supplemented diets. Regarding the main effect, dietary supplementation of Bet or Gly decreased (p<0.01) blood H:L ratio and feather CORT concentrations in aged laying hens under HS conditions.

## DISCUSSION

### Productive performance and egg quality

The current study revealed no significant effects of dietary supplementation of individual or combination of 0.65% Gly and 0.20% Bet on productive performance, including hen-day egg production, egg weight, egg mass, broken and shell-less eggs, FI, and FCR in aged laying hens under HS conditions. These findings contrast with those of previous studies reporting an improvement in productive performance by feeding diets supplemented with Bet to laying hens under HS conditions [6,14]. The reason for the inconsistency between previous studies and the current study is not clear because of the limited number of studies regarding dietary supplementation of Bet in aged laying hens under HS conditions. However, this variable result may be attributed to differences in the age of laying hens among studies. It has been suggested that laying hens become more vulnerable to HS with increasing age because aging facilitates the impairment in temperature regulation, antioxidant defense, nutrient utilization, and immune function [1,3]. Therefore, it is speculated that HS-induced reduction in laying performance following physiological impairments of aged laying hens cannot be fully mitigated by the current supplemental levels of dietary Bet.

Dietary Gly is recognized as a conditional essential AA in poultry, especially raised under stressful conditions because its requirements may be elevated owing to increased specific functions of Gly in the body [9,24]. A recent study revealed that increasing Gly supplementation from 0.4% to 1.6% in diets led to a linear improvement in feed efficiency of broiler chickens under HS conditions [24]. Furthermore, Nam et al [11] also reported that increasing Gly supplementation from 0.341% to 0.683% in diets linearly improved feed efficiency in 24-week-old laying hens under HS conditions. These previous findings are inconsistent with the current observation for no beneficial effects of dietary supplementation of 0.65% Gly on aged laying hens under HS conditions. The reason for this variable result may also be involved in the different age of poultry among studies because young laying hens and broiler chickens showed a positive effect of dietary Gly supplementation on productive performance, whereas such an effect was not identified in aged laying hens. Therefore, it may be inferred that the increase in dietary Gly requirement under HS conditions is greater for aged poultry than for young poultry, suggesting that more Gly supplementation in diets is required for aged laying hens under HS conditions. No impacts of dietary supplementation of individual Gly and Bet can also explain no synergistic interaction for productive performance in aged laying hens under HS conditions.

Impaired egg quality is frequently observed in laying hens raised under HS conditions [1,11]. Poultry exposed to HS exhibit increased panting, which leads to a respiratory alkalosis that compromises eggshell formation [1]. In addition, laying hens under HS conditions also experience with decreased FI and nutrient utilization in the body, thereby impairing egg quality [1]. However, no improvements in most egg quality were observed in this study. One possible reason may be that dietary Gly and Bet do not directly influence the specific hormonal and metabolic pathways involved in egg formation, such as calcium metabolism and shell gland function. Furthermore, increasing age of laying hens may reduce their physiological responsiveness for egg quality to dietary functional nutrients, making it challenging to observe improvements in egg quality. Interestingly, an interaction between dietary supplementation of Gly and Bet was found for eggshell color fan score. Dietary supplementation of Gly and Bet combinations improved eggshell color fan score compared with dietary supplementation of individual Gly and Bet, whereas no improvement was observed by feeding diets supplemented with Gly and Bet combinations compared with feeding the basal diet with no Gly and Bet supplementation. Although the reason for this interaction is difficult to explain, it may be related to the fact that Gly is a direct precursor of synthesizing protoporphyrin IX as an eggshell pigment, which is supported by our finding that dietary supplementation of Gly as the main effect tended to improve eggshell color fan score.

### Liver condition

Fatty liver hemorrhagic syndrome is commonly observed in laying hens, primarily due to enhanced fat accumulation with increasing age [17]. Moreover, HS promotes fat synthesis in the liver, and therefore, HS has been recognized as a possible factor that facilitates development of fatty liver hemorrhagic syndrome in laying hens [25].

In the current study, dietary Gly supplementation as the main effect mitigated the liver color score and MDA levels. Moreover, although the effects were not statistically significant, a decrease in other liver health measurements was also observed by dietary Gly supplementation. Similar results were also observed by Nam et al [11] who reported that increasing Gly supplementation in diets improved liver health by decreasing liver color score and fat concentrations in laying hens under HS conditions. In previous broiler studies, moreover, dietary Gly supplementation decreased MDA levels in the liver [12,24]. Accordingly, dietary Gly supplementation, regardless of Bet supplementation, may be recommended to improve liver health in aged laying hens under HS conditions. The beneficial effect of dietary Gly supplementation on improving liver health in poultry may be linked to the efficient conversion of Gly to Ser, which increases Ser availability in the body. The Gly is readily converted to Ser by serine hydroxymethyltransferase, and Ser can be used for the synthesis of phosphatidylethanolamine and phosphatidylcholine, which are major phospholipids involved in synthesis of very-low-density lipoproteins for exports of triacylglycerides from the liver [26]. Furthermore, dietary Gly may exert an antioxidant effect possibly because Gly is a structural AA for glutathione, which is a strong antioxidant [27]. Furthermore, involvement of Gly in one-carbon metabolism may be associated with improved liver health of aged laying hens under HS conditions. In poultry, the Gly cleavage system plays a crucial role in providing one-carbon units for uric acid synthesis in the liver, which is the primary route of nitrogen excretion in poultry [28].

Dietary Bet supplementation reduced liver MDA levels in this study, which is consistent with the finding of Alirezaei et al [29] who reported that dietary Bet supplementation decreased liver lipid peroxidation in laying hens. This antioxidant effect of Bet may be attributed to its ability to enhance the antioxidant defense in poultry under HS conditions [7,30]. Previous studies reported that dietary Bet supplementation enhanced the activity of key antioxidant enzymes, such as superoxide dismutase and glutathione peroxidase, in broiler chickens exposed to HS [29]. Furthermore, dietary Bet may improve the availability of AA, such as Gly and Cys, which are crucial for the synthesis of glutathione [8]. However, dietary Bet supplementation had no clear effects on improving other liver health measurements in this study. This result may be associated with the fact that the role of Bet as an osmolyte and methyl donor may prioritize its utilization for maintaining the cellular osmotic balance under HS conditions [7].

Despite the positive effects of individual Gly and Bet supplementation on decreasing lipid peroxidation in the liver, the absence of interactions between dietary Gly and Bet on other liver health measurements was found in this study, indicating no synergistic effects on improving liver health in aged laying hens under HS conditions. This observation may suggest that the mechanisms by which Gly and Bet alleviate oxidative stress and support liver health operate independently, and that combining them does not enhance their individual effects. A similar result was demonstrated by Won et al [12] reporting that dietary supplementation of 0.79% Gly and 0.20% Bet had no synergistic effects on liver health measurements in broiler chickens raised under HS conditions.

### Intestinal morphology and permeability

The adverse impacts of HS on intestinal morphology and barrier function in poultry have been documented [31,32]. Previous studies have reported that dietary Gly supplementation increased VH in broiler chickens under HS conditions [12,24]. A similar tendency for improving VH in aged laying hens under HS conditions was found by dietary Gly supplementation in this study. Dietary Bet supplementation has also shown to improve VH in broiler chickens in a previous study [33], whereas the current study found no such effect. Other research has reported that dietary Bet supplementation at the levels of 0.1% in broiler chickens or 0.2% in laying hens had no positive effects on VH under HS conditions [14,34]. Therefore, the effect of dietary Bet supplementation on VH in poultry under HS conditions remains inconclusive.

Higher VH:CD with increasing VH and decreasing CD has been considered an indicator of improved intestinal morphology in animals [35]. In this study, however, both individual and combined supplementation of Gly and Bet in diets increased CD in aged laying hens under HS conditions with no interactive effects being observed. Moreover, VH:CD did not differ among dietary treatments, possibly due to the relative increase in both VH and CD by dietary supplementation of Gly and Bet. Therefore, it should be noted that an increase in CD may reflect enhanced regeneration and turnover of intestinal villi, which is essential for maintaining intestinal health [36]. It appears that the roles of Gly in protein synthesis and cellular function as well as those in the osmoprotective properties of Bet may contribute to maintaining intestinal mucosa integrity [7,27].

Intestinal permeability, as indicated by TER values, was reduced by both individual and combination of dietary Gly and Bet in this study, suggesting that dietary Gly and Bet may enhance intestinal barrier function in aged laying hens under HS conditions. The improvement in intestinal barrier function is crucial for poultry health, particularly under HS conditions because HS is known to promote hypoxia and oxidative stress in intestinal tissues, leading to an impairment in intestinal barrier function [37]. Previous studies have demonstrated that dietary Gly supplementation improved intestinal health by its anti-inflammatory and cytoprotective actions in broiler chickens under HS conditions [12]. Similar improvements in intestinal barrier function have also been observed in broiler chickens fed diets supplemented with Bet [33]. This positive effect may be attributed to the physiological function of Bet, including its role as a methyl donor that promotes the proliferation of intestinal epithelial cells, its osmoprotective properties that improve the intestinal environment, and its antioxidant activity that alleviates oxidative damage. Furthermore, dietary Bet has been reported to improve microbial ecosystem in the intestinal tract of poultry under HS conditions [32,33], which may be indirectly associated with enhanced intestinal barrier function in poultry under HS conditions.

Despite the beneficial effect of individual Gly and Bet supplementation on intestinal barrier function, their combined supplementation resulted in little synergistic improvement. This observation may be attributed to the possibility that the maximal benefit on intestinal barrier function has already been achieved through individual supplementation, leaving limited potential for further improvement. Similar findings were reported by Won et al [12], in which combined supplementation of dietary Gly and Bet did not exhibit a synergistic effect on intestinal barrier function in broiler chickens under HS conditions. These results may suggest that the mechanisms through which dietary Gly and Bet enhance intestinal barrier function may reach a threshold effect when supplemented individually, such that their combination does not elicit an additive or synergistic response beyond the maximal effects achieved in aged laying hens under HS conditions.

### Stress indicators

The blood H:L is widely recognized as an indirect indicator of the stress response in poultry, with its elevated ratio reflecting increased stress levels [23]. In the current study, dietary supplementation of both Gly and Bet reduced blood H:L in aged laying hens under HS conditions. A similar reduction in blood H:L was observed in previous studies following dietary Gly supplementation in broiler chickens [12] and laying hens [11] under HS conditions. The effect of dietary Gly supplementation on decreasing blood H:L may be related to its anti-inflammatory and immunomodulatory properties [27]. Furthermore, the physiological role of Gly in the synthesis of phosphatidylserine, a phospholipid involved in cellular signaling and membrane function, may further support its possible stress-alleviating effects [38]. Furthermore, the ability of dietary Bet to reduce stress responses may be attributed to its osmoprotective properties, which aid in maintaining cellular function and integrity under stress conditions, thereby reducing the overall physiological stress burden [7]. In this study, feather CORT concentrations, another stress indicator, were also decreased by dietary supplementation of individual or combination of Gly and Bet in aged laying hens under HS conditions. Previous studies also reported decreased feather CORT concentrations in broiler chickens [12] and laying hens [11] following dietary supplementation of Gly or Bet. Therefore, these results suggest that dietary Gly and Bet can be potential functional nutrients to mitigate stress response in poultry under HS conditions.

Despite the individual benefits of Gly and Bet in reducing stress levels in this study, their combined supplementation did not exert a synergistic effect on both stress indicators. This result may suggest that although both dietary Gly and Bet can alleviate stress responses, their mechanisms of action may not be complementary enough to further enhance the reduction in stress levels. Moreover, this lack of synergistic effects may be associated with the possibility that the maximum stress-reducing capacity was already achieved through individual supplementation of dietary Gly and Bet, thereby minimizing any additional benefits on stress responses.

## CONCLUSION

Dietary supplementation of 0.65% Gly and 0.20% Bet improved liver health, intestinal barrier function, and reduced stress responses in aged laying hens under HS conditions. However, these beneficial effects were not conclusively reflected in productive performance and egg quality. Although a synergistic effect of combined supplementation of dietary Gly and Bet was anticipated, little additional benefit was observed for liver health, intestinal barrier function, and stress responses in aged laying hens under HS conditions.

## Figures and Tables

**Table 1:** Composition and nutrient content of basal diets (as-fed basis)

Items	Inclusion (%)			

	Basal	Glycine	Betaine	Glycine+Betaine
Ingredients				
Corn	61.56	61.56	61.56	61.56
Soybean meal (45% CP)	19.08	19.08	19.08	19.08
Corn gluten meal	4.42	4.42	4.42	4.42
Soybean oil	1.32	1.32	1.32	1.32
Monodicalcium phosphate	1.58	1.58	1.58	1.58
Limestone	9.45	9.45	9.45	9.45
DL-Methionine (98%)	0.21	0.21	0.21	0.21
L-Lysine HCl (98%)	0.19	0.19	0.19	0.19
L-Threonine (99%)	0.02	0.02	0.02	0.02
L-Isoleucine (99%)	0.02	0.02	0.02	0.02
L-Tryptophan (99%)	0.03	0.03	0.03	0.03
Glycine (99%)	0.00	0.65	0.00	0.65
Betaine (99%)	0.00	0.00	0.20	0.20
Alanine (99%)	0.75	0.00	0.75	0.00
Cornstarch	0.00	0.35	0.00	0.35
Celite	0.50	0.25	0.30	0.05
Salt	0.16	0.16	0.16	0.16
Choline Chloride	0.16	0.16	0.16	0.16
NaHCO_3_	0.10	0.10	0.10	0.10
Antioxidant	0.05	0.05	0.05	0.05
Vitamin premix^1)^	0.20	0.20	0.20	0.20
Mineral premix^2)^	0.20	0.20	0.20	0.20
Total sum	100.00	100.00	100.00	100.00
Calculated energy and nutrient content^3)^				
AME_n_ (kcal/kg)	2,750	2,750	2,750	2,750
Crude protein (%)	17.2	17.2	17.2	17.2
Digestible lysine (%)	0.78	0.78	0.78	0.78
Digestible methionine+cysteine (%)	0.69	0.69	0.69	0.69
Digestible methionine (%)	0.47	0.47	0.47	0.47
Digestible threonine (%)	0.51	0.51	0.51	0.51
Digestible tryptophan (%)	0.17	0.17	0.17	0.17
Digestible arginine (%)	1.60	1.60	1.60	1.60
Digestible isoleucine (%)	0.60	0.60	0.60	0.60
Digestible leucine (%)	1.52	1.52	1.52	1.52
Digestible valine (%)	0.67	0.67	0.67	0.67
Digestible glycine+serine (%)	1.29	1.94	1.29	1.94
Total calcium (%)	4.00	4.00	4.00	4.00
Available phosphorus (%)	0.37	0.37	0.37	0.37
Analyzed nutrient content				
Crude protein (%)	16.9	17.7	17.6	17.3
Total glycine (%)	0.68	1.18	0.69	1.17
Total serine (%)	0.84	0.78	0.84	0.76
Total glycine+serine (%)	1.52	1.96	1.53	1.93

1) Provided per kilogram of the complete diet: vitamin A, 11,700 IU (retinly acetate); vitamin D3, 3,600 IU; vitamin E, 27 IU (DL-α-tocopheryl acetate); vitamin K3, 2.7 mg (menadione dimethpyrimidinol); vitamin B1, 2.7 mg; vitamin B2, 6.3 mg; vitamin B6, 4.5 mg; vitamin B12, 18 μg; folic acid, 1.35 mg; biotin, 135 μg; niacin, 45 mg; pantothenic acid, 10.8 mg.

2) Provided per kilogram of the complete diet: copper, 7.35 mg; iron, 46.75 mg; manganese, 87.34 mg; zinc, 75.21 mg; chromium, 100 μg; selenium, 235 μg.

3) Calculated values from Rostagno et al [13].

**Table 2:** Effect of dietary supplementation of glycine and betaine on productive performance in aged laying hens under heat stress conditions

Treatments		Productive performance					

Glycine	Betaine	HD (%)	EW (g)	BS (%)	EM (g)	FI (g/d)	FCR (g/g)
0%	0%	90.6	60.0	0.63	54.2	102.3	1.89
	0.20%	89.6	59.6	0.83	53.4	102.0	1.91
0.65%	0%	90.8	58.8	0.71	53.5	102.8	1.92
	0.20%	89.6	59.4	0.72	53.0	101.8	1.92
SEM (n = 8)		0.82	0.58	0.174	0.71	0.57	0.027
Main effect							
Glycine							
0%		90.1	59.8	0.73	53.8	102.1	1.90
0.65%		90.2	59.1	0.72	53.2	102.3	1.92
SEM (n = 16)		0.61	0.41	0.158	0.50	0.40	0.019
Betaine							
0%		90.7	59.4	0.67	53.8	102.5	1.91
0.20%		89.6	59.5	0.77	53.2	101.9	1.92
SEM (n = 16)		0.61	0.41	0.158	0.50	0.40	0.019
Effect (p-value)							
Glycine		0.844	0.234	0.916	0.423	0.809	0.431
Betaine		0.128	0.864	0.329	0.378	0.281	0.656
Glycine×Betaine		0.854	0.415	0.359	0.780	0.523	0.631

HD, hen-day egg production; EW, egg weight; BS, broken and shell-less egg production rate; EM, egg mass; FI, feed intake; FCR, feed conversion ratio; SEM, standard error of the mean.

**Table 3:** Effect of dietary supplementation of glycine and betaine on egg quality in aged laying hens under heat stress conditions

Treatments		Egg quality							

Glycine	Betaine	Eggshell color				Yolk color	Haugh unit	Eggshell thickness (μm)	Eggshell strength (kg/cm^2^)

		Fan	L*	a*	b*				
0%	0%	10.4^ab^	50.1	20.1	28.0	8.5	85.8	377	3.60
	0.20%	9.6^b^	50.5	19.9	28.5	8.4	85.5	372	3.98
0.65%	0%	10.1^b^	50.2	20.1	28.5	8.4	87.2	378	3.79
	0.20%	11.4^a^	49.8	20.4	28.2	8.4	87.1	378	3.90
SEM (n = 8)		0.52	0.61	0.45	0.19	0.09	1.20	6.4	0.183
Main effect									
Glycine									
0%		10.0	50.3	20.0	28.2	8.5	85.6	375	3.79
0.65%		10.8	50.0	20.3	28.3	8.4	87.1	378	3.84
SEM (n = 16)		0.42	0.43	0.30	0.14	0.06	0.85	5.0	0.130
Betaine									
0%		10.3	50.2	20.1	28.2	8.5	86.5	378	3.70
0.20%		10.5	50.1	20.2	28.4	8.4	86.3	375	3.94
SEM (n = 16)		0.37	0.43	0.30	0.14	0.06	0.85	5.1	0.130
Effect (p-value)									
Glycine		0.094	0.589	0.537	0.600	0.532	0.230	0.499	0.768
Betaine		0.547	0.934	0.792	0.496	0.267	0.887	0.654	0.196
Glycine×Betaine		0.018	0.527	0.579	0.378	0.744	0.948	0.660	0.492

a,b Means in the same column with different superscripts are different (p<0.05).

SEM, standard error of the mean.

**Table 4:** Effect of dietary supplementation of glycine and betaine on liver health in aged laying hens under heat stress conditions

Treatments		Liver measurements				Serum measurements	
	
Glycine	Betaine	Color score	Hemorrhagic score	AEE (%)	MDA (μmol/mg protein)	AST (U/L)	ALT (U/L)
0%	0%	2.71	1.48	22.5	43.4	265	1.79
	0.20%	3.00	1.91	26.8	34.2	266	1.48
0.65%	0%	1.95	1.71	20.7	35.0	250	2.37
	0.20%	2.05	0.94	23.1	28.0	241	1.60
SEM (n = 8)		0.266	0.435	3.56	3.24	26.1	0.526
Main effect							
Glycine							
0%		2.86	1.69	24.7	38.8	266	2.04
0.65%		2.00	1.33	21.9	31.5	246	1.55
SEM (n = 16)		0.188	0.296	2.36	2.22	17.6	0.316
Betaine							
0%		2.33	1.60	21.6	39.2	258	1.56
0.20%		2.52	1.43	25.0	31.1	254	2.02
SEM (n = 16)		0.188	0.296	2.43	2.22	16.8	0.307
Effect (p-value)							
Glycine		0.004	0.389	0.409	0.025	0.412	0.266
Betaine		0.480	0.682	0.321	0.014	0.876	0.296
Glycine×Betaine		0.723	0.158	0.790	0.727	0.832	0.932

AEE, acid hydrolyzed ether extract; MDA, malondialdehyde; AST, aspartate aminotransferase; ALT, alanine aminotransferase; SEM, standard error of the mean.

**Table 5:** Effect of dietary supplementation of glycine and betaine on jejunal morphology and permeability in aged laying hens under heat stress conditions

Treatments		Intestinal characteristics			

Glycine	Betaine	VH (μm)	CD (μm)	VH:CD	TER (Ω/cm^2^)
0%	0%	600.4	73.4^b^	8.27	45.0^b^
	0.20%	676.4	92.5^a^	7.44	253.2^a^
0.65%	0%	704.6	90.4^a^	7.73	186.5^a^
	0.20%	795.1	90.5^a^	8.95	217.9^a^
SEM (n = 8)		59.42	4.31	0.758	27.37
Main effect					
Glycine					
0%		638.4	82.9	7.86	149.1
0.65%		749.8	90.5	8.34	202.2
SEM (n = 16)		42.02	3.04	0.536	18.65
Betaine					
0%		652.5	81.9	8.00	115.8
0.20%		735.8	91.5	8.19	235.5
SEM (n = 16)		42.02	3.04	0.536	18.65
Effect (p-value)					
Glycine		0.068	0.087	0.523	0.048
Betaine		0.167	0.033	0.798	<0.001
Glycine×Betaine		0.902	0.034	0.184	0.002

a,b Means in the same column with different superscripts are different (p<0.05).

VH, villus height; CD, crypt depth; VH:CD, villus height-to-crypt depth ratio; TER, trans-epithelial resistance; SEM, standard error of the mean.

**Table 6:** Effect of dietary supplementation of glycine and betaine on stress indicators in aged laying hens under heat stress conditions

Treatments		Stress indicator	

Glycine	Betaine	Blood H:L	Feather CORT (pg/mg)
0%	0%	0.38	16.14^a^
	0.20%	0.25	8.93^b^
0.65%	0%	0.27	8.58^b^
	0.20%	0.21	8.70^b^
SEM (n = 8)		0.024	0.954
Main effect			
Glycine			
0%		0.32	12.53
0.65%		0.24	8.64
SEM (n = 16)		0.017	0.675
Betaine			
0%		0.32	12.36
0.20%		0.23	8.81
SEM (n = 16)		0.017	0.675
Effect (p-value)			
Glycine		0.004	<0.001
Betaine		0.001	0.001
Glycine×Betaine		0.155	0.001

a,b Means in the same column with different superscripts are different (p<0.05).

H:L, heterophil to lymphocyte ratio; CORT, corticosterone; SEM, standard error of the mean.

## Data Availability

Upon request, the datasets of this study can be available from the corresponding author.
